# Impact of digital health interventions on medication adherence: A systematic review and meta-analysis

**DOI:** 10.1177/20552076251361593

**Published:** 2026-01-23

**Authors:** Catherine McCabe, Leona Connolly, Mary Hughes, Carmel Doyle, Arielle Weir, Margaret McCann, Maria Brenner

**Affiliations:** 1 8809 School of Nursing & Midwifery, Trinity College Dublin, Dublin, Ireland; 2 8797 School of Nursing, Midwifery & Health Systems, University College Dublin, Dublin, Ireland

**Keywords:** Digital health interventions, medication adherence, self-management, eHealth, mHealth

## Abstract

**Background:**

Digital health interventions for self-management have reported improvement in medication adherence, but further studies have reported that this is not sustained. Clarity is needed on the effectiveness of digital health interventions (DHIs) for medication adherence and the characteristics of successful DHIs.

**Objective:**

This review aimed to evaluate the effectiveness of DHIs versus standard care in supporting medication adherence in community-based adults.

**Methods:**

Studies of DHIs that included community-based adults (18 + years) that required prescribed medications and either used, or have access to, technology to manage their illness were included for review. A literature search was conducted across eight key databases. Studies were pooled in statistical meta-analysis using a random effects model. Pooled results were estimated by subgroup analyses defined by study design, intervention duration and intervention type (platform).

**Results:**

Thirteen studies were included in the final meta-analysis (*N* = 1320). There was no statistically significant effect of DHIs on medication adherence. Subgroup analyses showed no increase in medication adherence observed in the intervention group. However, a small significant increase in medication adherence was observed in the control group in studies of 6-month duration.

**Conclusions:**

The impact of DHIs on medication adherence is unclear. More rigorous clinical evidence is required to understand the impact of DHIs on medication adherence over longer durations, and further studies exploring the impact of platform type on medication adherence are required, with consideration given to the potential of Artificial Intelligence (AI)-driven diagnostics and analytics in the future.

## Introduction

Medication adherence can be defined as the extent to which medication taking behaviour is consistent with healthcare provider recommendations.^1,2^ Poor medication adherence is frequently reported as a main cause of unsuccessful pharmacotherapy and has been estimated to lead to 200,000 deaths annually in Europe.^3,4^ The World Health Organization reports that 50% of the global population is non-adherent to medication, and this number is increasing worldwide.^1,5,6^ Non-adherence has both medical and economic consequences, with adherence being associated with poorer clinical outcomes including: lack of treatment effectiveness; increased medical visits to general practitioners; increased hospitalisations; and increased morbidity and mortality.^5^ There are several major intervention methods that have been identified to influence medication adherence including education, behaviour and a multi-approach.

Increasingly, digital health interventions (DHIs), sometimes referred to as mHealth or eHealth, which utilise digital, mobile and wireless technologies for health, are being adopted into health systems to meet the growing need for accessible health coverage.^7^ These are digital solutions designed to improve health through prevention or health promotion, often through monitoring and communication,^8,9^ and can be delivered through personal computers and the use of applications for mobile technology, such as the iPad, Android tablets, smartphones and smartwatches.^10^ DHIs can offer the potential for self-management in the home by providing information, instruction, goal setting and self-monitoring through one or more approaches, such as video, audio, digital images or digital copies, to deliver educational and motivational content.

DHIs offer new methods for delivering healthcare and can have the potential to innovate healthcare services and enhance access, understanding and adherence to medication while allowing individuals to engage more closely and easily in their own care.^11,12^ They are also increasingly being used to support behaviour change and self-management of chronic conditions such as asthma, cardiac disease and chronic obstructive pulmonary disease.^13–17^

The use of DHIs provide people with relevant, individualised, motivational and educational material that may encourage, support and facilitate self-management, including medication adherence, and may reduce hospital readmission, acute exacerbations and costs.^18^ DHIs may facilitate self-management for people by providing low-cost educational and online resource materials that are always accessible.^10^

The rapid growth and uptake of mobile technologies, and the increasing use of smartphones, smartwatches, tablets and apps have made information more accessible to the general population and present opportunities to address healthcare problems, such as medication adherence.^19,20^ There are over six billion smartphone subscriptions worldwide, with this number expected to grow several hundred millions in the coming years.^21^ This could indicate that the continued development and growth of DHIs for self-management and medication adherence are a realistic and feasible healthcare strategy. Although many DHIs for self-management have reported improvements in management following the intervention, some studies have reported that these improvements are not sustained, regardless of whether the programmes were delivered via a DHI or delivered face-to-face.^13,22–24^ Given the vast number of digital health solutions currently available and the potential for DHIs to be integrated into routine medical care, there is an increasing need for evidence-based research demonstrating both the immediate and long-term impact of DHIs on health outcomes.^25^

This review evaluates the effectiveness of DHIs versus standard care (i.e. no DHI) in facilitating and supporting adherence to medication by adults in community-based (non-residential/institution-based) settings. The evidence may inform future research and technology related to medication adherence.

The objective of this study was to evaluate the effectiveness of DHIs versus standard care (i.e. no DHI) in facilitating and supporting adherence to medication by adults in community-based (non-residential/institution-based) settings.

## Methods

This review was conducted in accordance with the a priori protocol, which was registered with PROSPERO, the international systematic review registry funded by the National Institute for Health and Care Research (NIHR) (registration number CRD42024601867). Guidance from the Cochrane Handbook for Systematic Reviews of Interventions version 6.2 guided the structure for the methods used in this review.^26^ However, due to changes in institutional availability and the inclusion of randomised controlled trials (RCTs) and observational studies, JBI SUMARI software was used to conduct the meta-analysis.

### Inclusion criteria

#### Participants

Studies that included adult participants ( ≥ 18 years of age) taking medication for a health condition (both physical and mental health conditions) which requires prescribed medication were included. Studies were eligible if they included participants who were community-based (non-residential/institution-based) and either used, or have access to, technology, for example, a personal computer, tablet, smartphone or smart watch to manage their medications.

#### Intervention

We included studies that focused on DHIs, specifically computer and mobile interventions. This included remote and Web 2.0-based interventions delivered via technologies including personal computers and applications for mobile technology such as iPad, Android tablets and smart phones. We excluded studies that focused on monitoring devices such as telemonitoring/telehealth or assistive technologies because these studies involve the participation of more than one user, for example, the patient and the healthcare professional.

#### Comparators

This review considered studies that compared the intervention to standard care (i.e. no DHI). This included written, verbal and pictorial instruction and leaflets.

#### Outcomes

Included studies addressed the outcome of medication adherence by patients (e.g. self-report, pill count, prescription retrieval) or as defined from the included studies. Outcomes assessed at 1, 3 and 6 months were included as available from data in the included studies.

#### Types of studies

Study designs included were RCTs, quasi-experimental trials, case-control studies and cohort studies.

### Search strategy

A rigorous literature search was conducted across eight key databases: AMED - The Allied and Complementary Medicine Database, CINAHL Complete, Health Source: Nursing/Academic Edition, MEDLINE, APA PsycINFO, EMBASE, EBM Reviews - Cochrane Database of Systematic Reviews, EBM Reviews - Cochrane Central Register of Controlled Trials, EBM Reviews - Database of Abstracts of Reviews of Effects, and EBM Reviews - Health Technology Assessment. The key words and syntax were developed in the EBSCO Host platform and adapted to the other databases. All databases were searched from their inception to the present, with no language limit imposed. Non-English articles with English abstracts were reviewed. No restrictions based on year of publication were imposed. Both published and non-published articles were reviewed (append search terms). Reference lists of all primary studies and review articles were reviewed for additional references.

### Study selection

**Figure 1.**
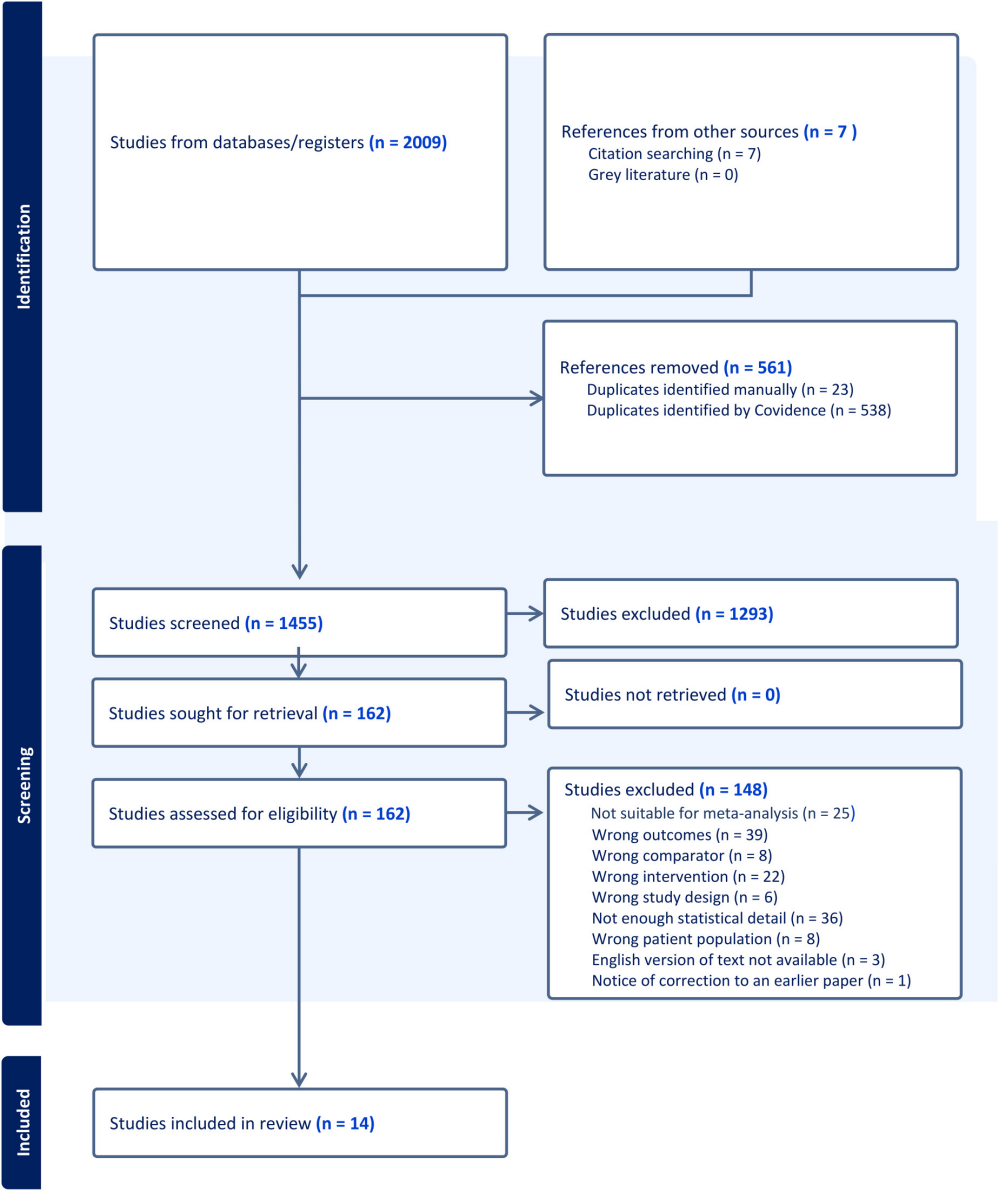
PRISMA flowchart.

Articles collected from the literature search were imported into the EndNote reference manager software programme and de-duplicated. They were then exported to Covidence Systematic Review software for screening and data extraction.^27^ Title and abstract screening were completed independently by two of the review team (AW, CMcC, MMcC, MB, MH and CD). Full-text study reports/publications were retrieved and independently reviewed by two study authors (two of: CMcC, LC, MMcC, MB, MH and CD) to identify studies for inclusion and record reasons for exclusion of ineligible studies. Disagreements were resolved through discussion to achieve consensus. We identified and excluded duplicates and collated multiple reports of the same study so that each study, rather than each report, was the unit of interest in the review. The results of the search and the study inclusion process are presented in a Preferred Reporting Items for Systematic Reviews and Meta-analyses flow diagram (Figure 1).^28^

PRISMA: Preferred Reporting Items for Systematic Reviews and Meta-analyses.

### Quality assessment

The quality of included studies was assessed independently by two members of the review team (two of: CMcC, LC, MH and CD) using the Effective Public Health Practice Project (EPHPP) quality assessment tool for quantitative studies.^29^ Included studies were graded as strong (no weak ratings), moderate (one weak rating) or weak (two or more weak ratings). Differences were discussed and adjudicated by a third member of the review team. Members of the review team were not blinded to authorship or journal.

### Data extraction

Data were extracted using a pre-designed extraction form, accessed in Covidence systematic review software and piloted on one study prior to data extraction. Data extracted included:

1. Methods: study design, total duration of study, number of study centres and location, study setting, withdrawals and date of study.
2. Participants: number, mean age, age range, gender, condition, diagnostic criteria, inclusion criteria and exclusion criteria.
3. Interventions: intervention, comparison, concomitant medications and duration of intervention.
4. Outcomes: primary outcome specified and collected, and time points reported, method to measure adherence (e.g. electronic medication event monitoring, pharmacy refills, pill counts and self-report).
5. Notes: funding for trial and notable conflicts of interest of trial authors.

Two review authors (two of: CMcC, LC, MH and CD) independently extracted outcome data from included studies. Disagreements were resolved by consensus among authors (CMcC, LC, MH or CD). A second review author (either CMcC, LC, MH or CD) conducted a spot-check of study characteristics for accuracy against the report.

### Data synthesis

Studies were pooled in statistical meta-analysis using JBI SUMARI^30^ using a random effects model.^31^ Effect sizes are expressed as standardised mean differences and 95% confidence intervals. Pooled results were also estimated by subgroup analyses defined by study design (RCT vs observational), intervention duration (1 months, 3 months or 6 months), intervention type (platform), study type (RCT or observational) and outcome measure (self-report or objective).

### Sensitivity analysis and investigation of heterogeneity

Sensitivity and heterogeneity analyses were conducted to investigate statistical variation and differences in effect size and strength of conclusions. Decisions regarding the inclusion of studies were informed by the χ^2^ (Q) test and *I*^2^ statistical tests. A *p* value of less than .10 or an *I*^2^ of more than 50% suggested substantial heterogeneity. A fixed-effects model was compared with outcomes of the random effects model as part of sensitivity analysis to assess robustness.

## Results

### Study characteristics

Two thousand and nine references were imported for screening. Five hundred and sixty-one duplicates were identified and removed. Of these, 1455 studies were screened against title and abstract with 1293 studies excluded at this stage. In total, 162 studies were assessed for full-text eligibility and 148 studies were excluded for not meeting eligibility criteria (Appendix I, Table 1). Fourteen studies were included for analysis. However, one was excluded following tests of heterogeneity. Thus, the final meta-analysed outcomes are derived from 13 studies. Reasons for exclusion were; not suitable for meta-analysis (*n* = 25), wrong outcomes (*n* = 39), wrong comparator (*n* = 8), wrong intervention (*n* = 22), wrong study design (*n* = 6), not enough statistical detail (*n* = 36), wrong patient population (*n* = 8), English version of text not available (*n* = 3), notice of correction to an earlier paper (*n* = 1) (Figure 1). Studies retrieved were conducted between 2014 and 2021, with most studies being conducted between 2015 and 2018 (8/14).

### Methodological quality

#### Critical appraisal

The EPHPP was used for the critical appraisal of studies. This tool contains eight sections which are graded strong, moderate to weak and then allocated a global score based on these. The areas assessed are selection bias, study design, confounders, blinding, data collection methods, withdrawals and drop puts, intervention integrity and methods of analysis. Of the 14 studies included in the meta-analysis, one was of strong quality,^32^ 10 were of moderate quality,^33–42^ and three were of weak quality (Appendix II, Table 2).^43–45^

#### Characteristics of included studies

Of the 14 studies that met inclusion criteria, the majority (8/14) were conducted in the United States. One was conducted in Canada, the Netherlands, Pakistan, Ghana, New Zealand, and China, respectively. The mean sample size was 102 participants, ranging from 19 to 478 participants, with a total sample size of 1424. Thirteen studies provided mean ages of participants which ranged from 26 to 68 years with an overall mean age of 53 years. The gender profile of participants was 64% male across studies. The illness profiles of participants included seven studies of participants with cardiovascular illness (atrial fibrillation x 2, coronary heart disease (CHD) x 2, cerebrovascular accident/coronary artery disease (CVA/CAD) x 1, hypertension x 1 and hypertension plus diabetes x 1), two studies in participants with Diabetes, one study in a population of stroke patients, one study in a human immunodeficiency virus (HIV) population, one study among kidney transplant recipients and one study conducted among smokers. Most interventions were 3 months in duration (6/14), while (4/14) lasted for 1 month and (3/14) lasted for 6 months. Five studies conducted the intervention using mobile apps (5/14), and five studies used a text messaging platform (5/14). Two interventions were web-based, and one combined a web and text messaging platforms. One intervention provided an electronic medication tray in conjunction with a text messaging service. Most studies (12/14) used a self-reported measure of medication adherence, while two studies (2/14) used an objective measure. Statistically significant difference in medication adherence was observed in 7/14 studies, four of which were RCTs (Appendix III, Table 3).

## Review findings

### Effect of DHIs on medication adherence

Fourteen studies evaluated the effect of a DHI on medication adherence. Following sensitivity analysis, one study was removed as it introduced substantial heterogeneity leaving 13 studies in the final analyses. Compared with control groups (usual care), there was no increase in medication adherence for those in the intervention group (–0.21 [−0.02, 0.43], *p* = .07). Tests of heterogeneity indicate substantial statistical heterogeneity among studies (χ^2^ = 47.11, *df* = 13, *p* = 0), supported by the *I*^2^ of 73% (Appendix IV, Figure 2). Examination of the plot suggest bias may be attributed to one study.^35^ Sensitivity analysis confirmed that excluding this study from the pooled analysis reduced statistical heterogeneity to (*p* = .03, *I*^2^ = 47), and adopting this approach resulted in a significant but small effect size (0.28 [0.12, 0.44], *p* = 0). For this reason, this study was removed from further analyses, and all subsequent findings are derived from the remaining 13 studies (*N* = 1320) (Figure 2).

**Figure 2.**
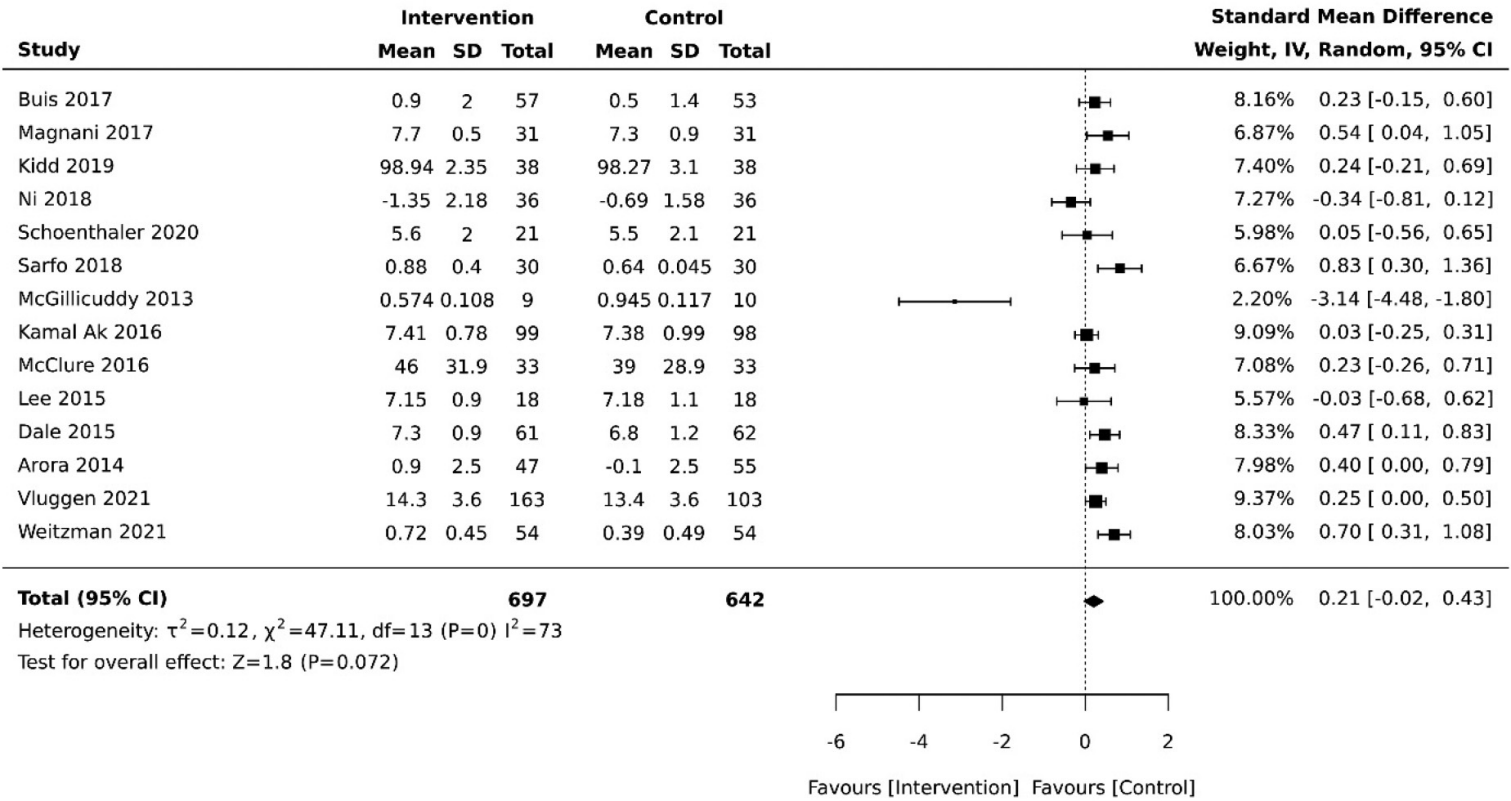
Summary effect of DHIs on medication adherence vs control group.

DHI: digital health intervention.

**Figure 4.**
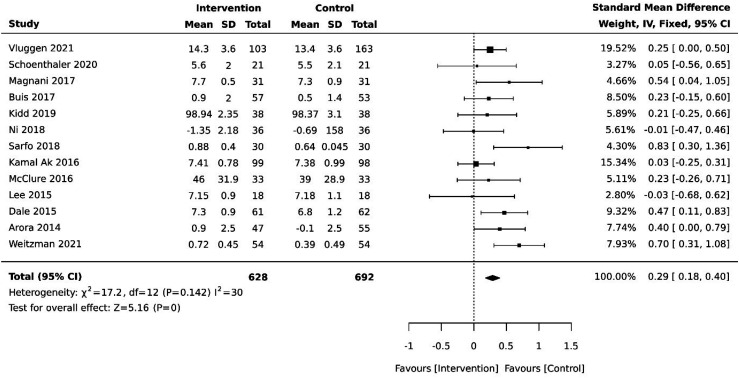
Sensitivity analysis; fixed-effects model.

A fixed-effects model was conducted to further assess sensitivity, resulting in moderate heterogeneity (*I*^2^ = 30, *p* = .14) with a small significant increase in medication adherence in the control condition (0.29 [0.18, 0.40], *p* = 0) (Appendix V, Figure 4).

**Figure 5.**
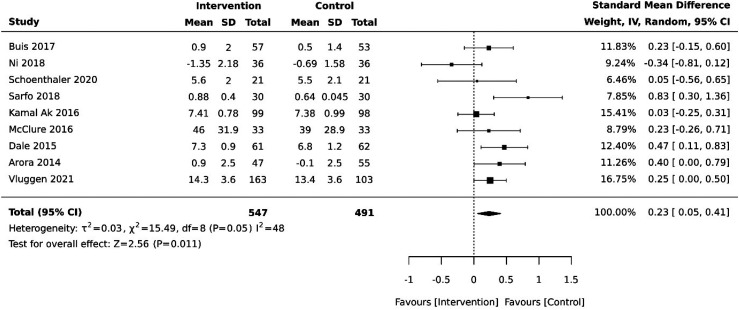
Summary of effect size; randomised controlled trials.

**Figure 6.**
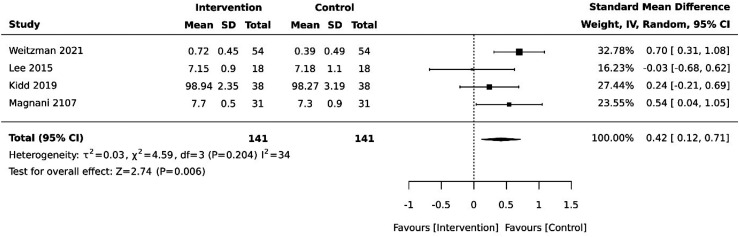
Summary of effect size; observational studies.

Subgroup analysis of medication adherence outcomes in RCTs (*n* = 9) compared with observational studies (*n* = 4) shows that there is a statistically significant small effect size difference in medication adherence favouring the control group of *p* = .01 (0.23 [0.05, 0.41] and *p* = .006 (0.42 [0.12, 0.71] respectively) (Appendix VI, Figures 5 and 6).

**Figure 7.**
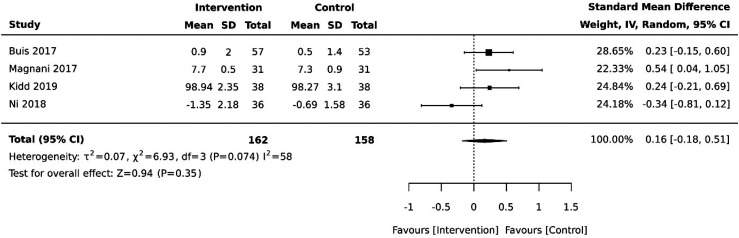
Summary effect for interventions of 1-month duration.

**Figure 8.**
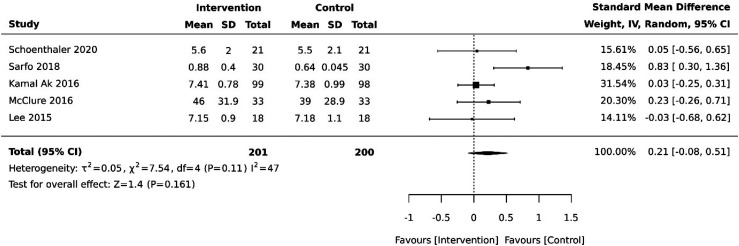
Summary effect for interventions of 3-month duration.

**Figure 9.**
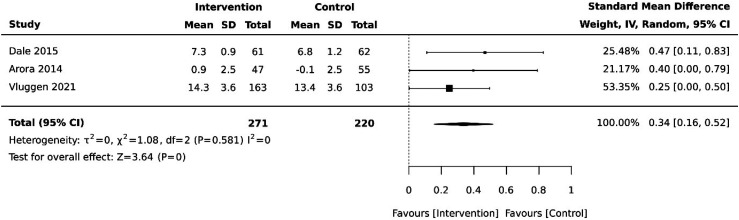
Summary effect for interventions of 6-month duration.

Further subgroup analysis was conducted to assess medication adherence outcomes at 1 month, 3 months and 6 months. Weitzman et al.^45^ were excluded from this analysis as the intervention ran for a 6-week duration. There was no effect of DHIs on medication adherence in studies that reported on interventions of 1 month (*n* = 4) (0.16 [−0.18, 0.51], *p* = .35) or 3 months (*n* = 5) (0.21 [−0.08, 0.51], *p* = .16). However, there was a moderate increase in medication adherence in studies reporting intervention of 6 months (*n* = 3) in duration (0.34 [0.16, 0.52], *p* = 0) (Appendix VII, Figures 7, 8 and 9).

**Figure 10.**
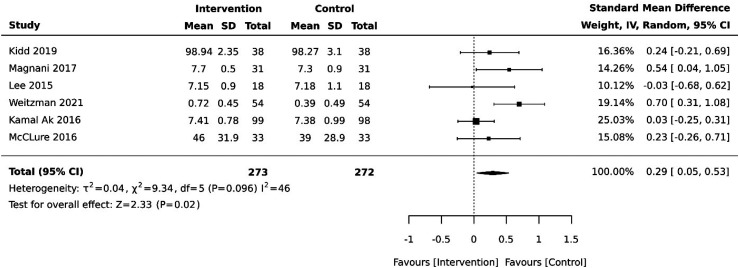
Summary effect for mobile apps.

**Figure 11.**
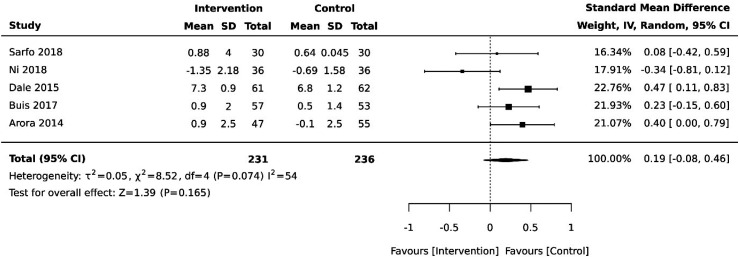
Summary effect for text messaging platforms.

**Figure 12.**
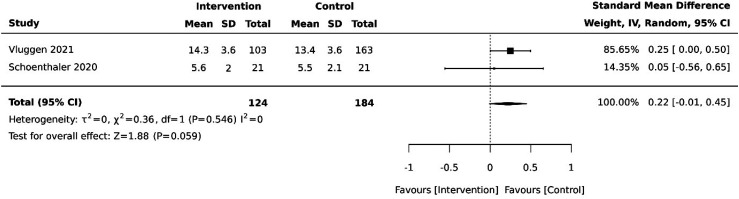
Summary effect for web-based interventions.

Six studies reported medication adherence outcomes for interventions that used mobile apps.^36,37,41,42,44,45^ A further five reported on interventions that used text messaging platforms,^34,38–40,43^ and two studies reported on web-based interventions.^32,33^ A small but non-significant effect size favouring the control group was observed in subgroup analysis of studies of interventions using mobile apps (0.21, [−0.08, 0.51], *p* = .11). A small significant effect in favour of the control condition was observed for studies that used text messaging platforms (0.29 [0.05, 0.53], *p* = .02). Two studies reported adherence for web-based interventions and pooled analysis shows a small significant effect favouring the control condition (0.22 [−0.01, 0.45], *p* = .059) (Appendix VIII, Figures 10, 11 and 12). Summary statistics for subgroup analyses are presented in the Summary of Findings table (Table 1).

**Table 1.** Summary of findings.

Population: Comparator: Intervention: Outcome:	Adult participants ( ≥ 18 years of age) taking medication for a health condition (both physical and mental health conditions) which require prescribed medication were included. Digital health interventions Standard care Medication adherence					
Outcomes	Control *M* (*SD*)	Intervention *M* (*SD*)	Effect size [95% CI]	Number of participants (studies)	Quality of evidence (EPHPP)	Comments
Medication adherence	12.8 (23.3)	13.6 (25.1)	0.28 [0.12, 0.44]	1320 (13)	Strong *n* = 1 Moderate *n* = 10 Weak *n* = 3	No increase in medication adherence in intervention condition. Overall, studies were of moderate quality.
*Medication adherence grouped by study design*						
RCTs	8.6 (11.8)	9 (14.1)	0.23 [0.05, 0.41]	1038 (9)	Strong *n* = 1 Moderate *n* = 7 Weak *n* = 1	A small significant increase in medication adherence observed in the control condition in RCTs and observational studies.
Observational	29.2 (42.3)	29.6 (42.4)	0.42 [0.12, 0.71]	282 (4)	Strong *n* = 0 Moderate *n* = 2 Weak *n* = 2	RCT studies were predominantly of moderate quality. Observational studies were equally of moderate and weak quality.
*Adherence grouped by intervention duration*						
1 month	25.1 (41.5)	24.7 (41.4)	0.16 [−0.18, 0.51]	320 (4)	Strong *n* = 0 Moderate *n* = 3 Weak *n* = 1	
3 months	11.4 (17.1)	12.6 (19.7)	0.21 [−0.08, 0.51]	401 (5)	Strong *n* = 1 Moderate *n* = 3 Weak *n* = 1	
6 months	8.2 (6.2)	9.3 (6.1)	0.34 [0.16, 0.52]	431 (3)	Strong *n* = 0 Moderate *n* = 3 Weak *n* = 0	A moderate increase in medication adherence was observed in the intervention condition at 6 months only, among three studies of moderate quality.
*Adherence grouped by intervention platform*						
Mobile apps	22.5 (34.1)	23.5 (34.9)	0.29 [0.05, 0.53]	545 (6)	Strong *n* = 0 Moderate *n* = 4 Weak *n* = 2	A small significant increase in medication adherence was observed in the control conditions where text messaging and web-based platforms were used.
Text messaging	1.9 (3.4)	2.2 (3.6)	0.19 [− 0.08, 0.461]	467 (5)	Strong *n* = 0 Moderate *n* = 4 Weak *n* = 1	
Web-based	12.5 (4.3)	12.8 (4.7)	0.22 [−0.01, 0.45]	308 (2)	Strong *n* = 0 Moderate *n* = 1 Weak *n* = 1	

CI: confidence interval; EPHPP: effective public health practice project quality assessment tool; RCT: randomised controlled trial; SD: standard deviation.

As most studies measured medication adherence using self-reported questionnaires (12/13) it was not possible to conduct a subgroup analysis comparing self-reported with objectively measured adherence. However, 50% (6/12) of studies that used self-reported medication scales reported a significant increase in medication adherence in intervention groups compared with 0% (0/1) of studies that measured adherence objectively.

## Discussion

The purpose of this study was to assess the impact of DHIs on the outcome of medication adherence. A meta-analysis of the 13 studies included showed no statistically significant effect of DHIs on medication adherence compared with usual care. However, there was evidence of a higher level of medication adherence in the control group. Examination of RCTs compared with observational studies showed a similar trend with no increase in medication adherence observed in the intervention group. There was no increase in medication adherence in studies that were 1 month or 3 months in duration. However, in studies of 6-month duration a moderate increase in medication adherence was observed. In addition, the type of platform used in interventions (web-based, mobile apps or text messaging services) had no significant impact on medication adherence.

There was no evidence of an increase in medication adherence in the intervention group. In fact, the data indicate that overall, there was a small significant increase in adherence to be seen in the control group. Two studies favoured the intervention^34,42^; however, neither were statistically significant, and the wide confidence intervals indicate a lack of precision. However, some commonalities exist, namely in the inclusion of an educational component. For instance, Ni et al. (2018) developed a text messaging app to send daily medication reminders and weekly educational material to CHD patients specifically to increase medication adherence. Whereas Lee et al. (2015) designed an educational mobile application focusing on improving knowledge relating to diet and medication adherence for the management of cardiovascular disease, without medication reminders.

Although the removal of one study reduced the level of heterogeneity from 73% to 47%, moderate heterogeneity between studies still exists indicating a high level of variation between studies. This variation may be the result of differences between interventions. For example, clinical differences between studies included differences in how interventions were designed and implemented (platforms used and duration of studies) and differences between study populations in terms of age range and illness profile. Additionally, this was compounded by the varying definitions of optimal adherence used between studies. This variation between studies may explain this interesting finding.

Overall, the quality of the evidence was moderate (*n* = 9). However, some sources of bias were observed. For example, five studies were graded as weak in terms of controlling for confounding bias and a further five studies did not implement blinding. Four studies scored a weak rating in reporting withdrawals and drop-outs, and three studies showed evidence of selection bias. Confounding can arise from over or under adjustment of variables which can obscure the true effect of an intervention.^46^ In the included studies, the majority did not adjust the alpha level to account for multiple comparisons, thus this introduces the risk of Type II error in the data. In addition, the lack of blinding in five out of 13 studies may increase the risk of incorrect estimations of the effect of interventions.^47^

Further examination of the data also showed that when studies were analysed in terms of study design (RCTs [*n* = 9] vs observational studies [*n* = 3]), there was a small significant increase in medication in the control group. Analysis of heterogeneity indicates that there was a greater level of heterogeneity within RCTs (*I*^2^ = 48%) compared with observational studies (*I*^2^ = 34%), albeit still moderate in both groups. Among the RCTs examined, one study was classed as weak, seven studies were classed as moderate, and one study was classed as being of strong quality. Within the observational studies, two were classed as weak and two as moderate.

The length of the interventions included was also examined through subgroup analysis. Although there were no significant differences in adherence at 1 month and 3 months, there was a significant increase in medication adherence in the control group at 6 months. The differences in results between the three subgroups might be explained by the varied levels of heterogeneity between the 1-month group (*I*^2^ = 58%), 3-month (*I*^2^ = 47%) and the 6-month group which demonstrated considerably lower levels of heterogeneity (*I*^2^ = 0%).

Mobile apps were the adopted DHI in five papers with the aim of improving healthcare accessibility, efficiency and patient engagement relating to a variety of conditions or health concerns: atrial fibrillation, schizophrenia, HIV, CVA (cerebrovascular accident), CAD (coronary artery disease), smoking. These types of apps leverage smartphones, wearables and AI to provide real-time monitoring, personalised care and health education.^48^ Similarly, text messaging platforms were also adopted in five papers. These are heralded as a powerful and cost-effective DHI, improving patient engagement, adherence and health outcomes widely used in preventive care, chronic disease management, medication adherence and behaviour change programs.^49^ In this review, text messaging platforms were the chosen DHI for conditions relating to stroke, hypertension, CHD, kidney disease and diabetes. Web-based DHIs were adopted in two studies with a combination of both web and text messaging used in another study. These DHIs addressed such health-related issues as hypertension, diabetes and smoking. Web-based DHIs use websites, online portals and web applications to deliver healthcare services, education and self-management tools. Though there is currently no gold standard for DHIs to support medication adherence, the World Health Organisation (WHO) Recommendations on Digital Interventions for Health System Strengthening assert that these interventions enhance accessibility, efficiency and patient engagement while supporting preventive care, chronic disease management and mental health services.^50^ All of the DHIs included in this review measured adherence via participant self-report and all involved an educational component prior to engagement with the specific DHI.

As DHIs use has grown in healthcare and with an increased attention it is important to establish the relevance of this review to the DHI landscape. While there was evidence of a higher level of medication adherence in the control group it is plausible this was merely due to participant engagement in a research study. Nevertheless, with medication non-adherence a significant problem, DHIs have the potential to improve medication adherence, with this review demonstrating a moderate increase in studies of 6-month duration. In particular, those individuals with chronic illness such as those delineated in this review (e.g. diabetes, hypertension or CHD) are likely to benefit from improved medication adherence where these interventions are used in combination with other treatments.

## Limitations

Several limitations were encountered during this review. Most notably there was considerable methodological and statistical heterogeneity among included studies. This was evident in the variation in the definitions and measures of medication adherence, variation in DHI design and lack of statistical consistency, for example, consistency in correcting for multiple comparisons among studies. The latter may have thus led to an increased risk of error in the reporting of outcomes. Additionally, the primary aim of all included studies was not solely to enhance medication adherence, but studies also sought to improve health literacy, self-management, quality of life and clinical and health outcomes. As such, interventions used varying degrees of educational content and elements of behavioural change, leading to considerable methodological differences. The use of self-reported measures of medication adherence may have increased to potential for recall bias. Similarly, the use of blinding was absent in several studies which detracted from the overall quality of studies included, as did the treatment and reporting of participant withdrawals. Indeed, out of the 13 studies included in the final analysis just one was of strong quality, while the majority were of moderate quality (*n* = 9) and three were of weak quality. There were also a limited number of studies available for review.

Finally, this review excluded studies which focused on monitoring devices or assistive technologies that involved the participation of more than one user, for example, the patient and the healthcare professional. As such, there is a lack of insight on the impact of DHIs which incorporate clinician monitoring and feedback. Some prior research suggests that an initial period of 3 months is needed to establish a pattern of adherence,^51^ highlighting the possible role of clinician involvement for tailoring of DHIs. These limitations indicate the need for a rigorous clinical study to add to the existing body of evidence.

## Conclusions

This meta-analysis of the available data found no significant improvements in medication adherence following DHIs except where interventions were at least 6 months long, suggesting that duration of DHIs may be an important factor in the design of such interventions for the purpose of enhancing medication adherence. Overall, the quality of the included studies was moderate, and these findings may reflect the lack of homogeneity in DHI study methodologies and reporting, and clinical differences in how medication adherence was defined and measured. This highlights a need for standardisation in research methodologies in this area. Mobile app-based interventions were the most commonly used format for DHIs, followed by text messaging interventions, with interventions delivered through web-based platforms less commonly assessed. This may be reflective of the current trajectory of digital technology and digital usage trends and the popularity of mobile apps. There is a need for more rigorous clinical data to add to the body of knowledge that currently exists regarding the efficacy of DHIs for medication adherence and we have made a number of recommendations and comments regarding future directions of research of this topic.

## Recommendations for practice or policy

DHIs offer a range of technology driven solutions for medication adherence. The following recommendations for practice and policy should be considered. Patient and public involvement and stakeholder engagement are key in development and evaluation of DHIs to boost applicability and appropriateness for the end user taking into consideration digital health literacy. Healthcare professional's competency training in digital health literacy is also required to drive quality DHI development. Furthermore, equitable access for diverse populations, considering such issues as cultural appropriateness and language barriers, needs to be considered. National health policy requires digital health strategies and frameworks, currently not available in some countries. These should support a funding model for the development of sustainable and cost-effective DHIs.

### Future directions for research

As DHIs are being developed at an increasing pace it is important to consider the direction for future research. Interestingly, the platform type adopted in the studies had no significant impact on the outcome. Therefore, further studies exploring platform types will be necessary to establish if this may impact outcomes for the end user. Overall, the applicability of DHIs will be largely influenced by AI-driven diagnostics and analytics into the future. It will be important to establish how they can be embedded within existing and new electronic health record systems promoting seamless care. With text messaging an adopted DHI, future studies must consider the use of AI-driven chatbots for an interactive element and the possibility of two-way text function enabling real-time communication between the patient and provider. Assessment of DHI adverse effects is important and should be considered when developing such interventions and undertaking research evaluation. Furthermore, DHIs need rigorous clinical trials with exploration over longer durations and in other groupings such as the younger and older population where medication adherence can be more challenging.

### Possible challenges

While DHIs are considered beneficial, they also come with challenges that can hinder their use and effectiveness. It is essential that data privacy, consent and security are considered, and specific regulations are complied with. End users require effective data privacy and security in compliance with healthcare regulations and also provision of transparent data usage policies with explicit consent for data collection or sharing. Messaging personalisation is key, although must be balanced with automation and individually tailored advice. Furthermore, there are portions of the world population who have no access to internet and limited smartphone usage so access will be a key factor in development of DHIs into the future. Additionally, poor digital literacy will be a factor in implementation. In using DHIs, there may also be unexpected negative outcomes, all too often not considered, resulting in a need for vigilance in assessing adverse effects if they are to occur such as over engagement, cyber bullying and misinformation. A lack of regulation in this space requires attention and clear guidance on development of DHIs such as that published by the WHO. This will be fundamental in standardising formats for easy integration with other systems such as electronic health records. Scientific evidence of success will be a key factor in further development of DHIs expanded on in our recommendations for research. While DHIs have enormous potential, consideration of these challenges is critical for development and successful implementation. It is clear that collaboration between individuals, healthcare professionals, providers and policy makers is necessary in developing effective DHIs.

## Figures and Tables

**Figure 2.**
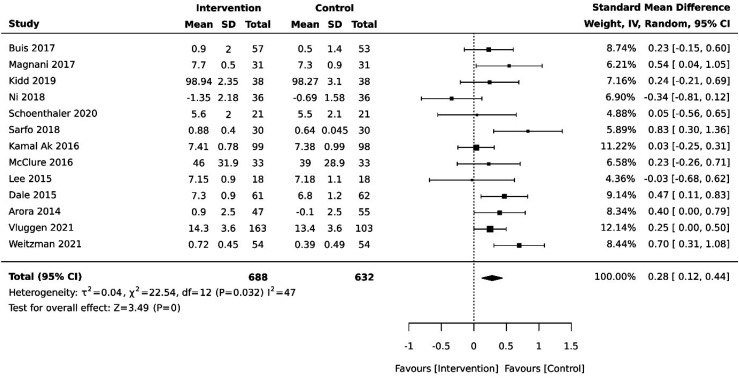
Impact of DHIs on medication adherence.

DHI: digital health intervention.

## Abbreviations

DHI: Digital health intervention
CVA: Cerebrovascular accident
CAD: Coronary artery disease
CHD: Coronary heart disease
HIV: Human immunodeficiency virus
PPI: Patient and public involvement
WHO: World Health Organisation

## Appendices

### Appendix I: Studies excluded on full text

**Table 1.** Studies excluded on full text.

Authors	Year published	Reason for exclusion
Aguas M; Del Hoyo J. Faubel R; Munoz D; Dominguez D; Bastida G; Navarro B; Cordon G; Barrios A; Valdivieso B; Correcher M; Nos P	2018	Wrong intervention
Aikens, James E.; Rosland, Ann-Marie; Piette, John D.	2015	Wrong intervention
Aikens, James E.; Zivin, Kara; Trivedi, Ranak; Piette, John D.	2014	Wrong outcomes and intervention may not be suitable
Ajcevic, Milos; Accardo, Agostino; Furlanis, Giovanni; Naccarato, Marcello; Caruso, Paola; Polverino, Paola; Manganotti, Paolo; Marsich, Alessandro	2021	No control condition and wrong outcomes
Akinbosoye, Osayi; Taylor, Darius; Jiang, Jenny; Taitel, Michael; Orr, Greg	2016	Wrong outcomes
Allsion, Matt; McMorris, Michael; Patel, Dhiren; Burton, B. Stephen	2019	Conference abstract only - no info re the intervention and adherence reported only as percentages
Arnavielhe, S.; Fonseca, J.; Silva, J. da; Fuentes Perez, M.; Huerta Villabolos, Y. R.; Just, J.; Kuna, T. P.; Schmidt-grendelmeier, P.; Tomazic, P.; Papadopoulos, N.; Bosnic-anticevich, S.; O'Hehir, R.; Zernotti, E.; Melen, E.; Hellings, P.; Dykewickz, M.; Naclerio, R. N.; Wallace, D. VDarsow, U.; De Blay, F.; Palkonen, S.; De Carlo, G.; Dedeu, T.	2018	Emphasis on symptom assessment, not medication adherence
Arora, Sanjay; Menchine, Michael; Peters, Anne L.; Agy, Chad	2012	Wrong outcomes
Atreja, Ashish; Szigethy, Eva; Otobo, Emamuzo; Chang, Helena L.; Keefer, Laurie; Rogers, Jason; Kohli, Akshay; Ullman, Thomas A.; Marion, James F.; Cohen, Benjamin L.; Maser, Elana; Itzkowitz, Steven; Colombel, Jean Frederic; Sands, Bruce E.	2018	Wrong outcomes
Bakker, Mirthe; Sloots, Joanne; Van Ommeren, Clara; Kleberger, Alexandra; Linssen, Gerard; Tabak, Monique; Effing, Tanja; Grinovero, Martijn; Van Der Palen, Job; Lenferink, Anke	2019	No control condition
Ballout, Ghada; Al-Shorbaji, Najeeb; Shishtawi, Amin; Abuzabaida, Fathia; Shahin, Yousef; Khader, Ali; Zeidan, Wafa'a; Turki, Yassir; Al-Natour, Ahmad; Saadeh, Rawan; Hababeh, Majed; Seita, Akihiro	2017	Wrong outcomes
Berges, Bethany Mooney; Cambareri, Christine; Takvorian, Samuel U.; Serpa, Mike; Shulman, Lawrence N.; Bekelman, Justin E.; Rendle, Katharine A.; Argon, Jesse; Rosin, Roy	2019	No statistical detail
Bonacini, Maurizio; Kim, Yoona A.; Reichert, Marcel; Travis, Ellen; Virdi, Naunihal; Raja, Praveen; Savage, George	2016	Wrong intervention
Brath, Helmut; Morak, JÃ¼rgen; KÃ¤stenbauer, Thomas; Modre-Osprian, Robert; Strohner-KÃ¤stenbauer, Hermine; Schwarz, Mark; Kort, Willem; Schreier, GÃ¼nter	2013	Wrong intervention - intervention included phonecalls from researchers to participants with low adherence to motivate adherence
Buis, Lorraine R.; Roberson, Dana N.; Kadri, Reema; Plegue, Melissa A.; Danak, Shivang U.; Guetterman, Timothy C.; Richardson, Caroline R.; Rockey, Nicole G.; Johnson, Melanie G.; Choe, Hae Mi	2020	Wrong intervention - intervention included follow up calls by pharmacists in response to high bp/low adherence readings
Burton, S.; Srivastava, U.; Patel, D.; Rasulnia, M.	2018	Wrong intervention
Car, Josip; Huang, Zhilian; Tan, Eberta; Lum, Elaine; Sloot, Peter; Boehm, Bernhard Otto	2020	References is a notice of correction to an earlier paper
ChiCtr,	2018	Reference is a link to a trial registry - protocol for the trial only
Ciavarella, N.; Virgilio, M.; Dirienzo, G.; Renna, L.; Pansini, N.	2012	Not adequate statistical detail
Costa, Elisio	2020	Wrong topic
Daly, Anne	2017	Wrong topic
Das Gupta, Debasree; Chandler, Kerianne; Patel, Amit; King, Andrew; Saxena, Deepak; Trivedi, Poonam; Patel, Krupali; Raval, Devang; Koizumi, Naoru	2020	Wrong intervention - involved live calls from study researchers as part of intervention
Davidson, Tatiana; Favela, April; Villamizar-Escobar, Jorge; Brunner-Jackson, Brenda; Mueller, Martina; Treiber, Frank	2015	Wrong outcomes - does not report on medication adherence as an outcome
De Keyser, Heather E. H.; Anderson, William C.; Szefler, Stanley J.; Kaye, Leanne; Gondalia, Rahul; Theye, Ben; Stempel, David A.	2020	Paediatric population
del Hoyo, Javier; Nos, Pilar; Faubel, Raquel; Munoz, Diana; Dominguez, David; Bastida, Guillermo; Navarro, Belen; Barrios, Alejandra; Valdivieso, Bernardo; Correcher, Marisa; Aguas, Mariam	2018	Not enough statistical detail for analysis
DeVito Dabbs A, Dew M. Myers B. A. Song M. K. Li R. Zomak R. Pilewski J. M. Bermudez C. A.	2014	Wrong outcomes - unable to extract medication adherence data from composite treatment adherence data
Dew, M.; Li, R.; Pilewski, J. M.; DeVito Dabbs, A.; Myers, B. A.; Song, M. K.; Zomak, R.; Bermudez, C. A.	2014	Wrong outcomes
Drake, Richard; Haddock, Gill; Nordentoft, Merete; Ainsworth, John; Lewis, Shon	2018	Wrong intervention
Du, Xin; Ma, C.; Jornten Karlsson, M.; Ryden, A.; Ahlqvist, M.; Sunden, M.; Xu, Y.; Guo, J.; Li, J.; Mei, X.; Han, L.; Wang, J.; Shen, Z.; Karlson, B. W.	2017	Wrong outcomes
Du, X.; Ma, C.; Jornten-Karlsson, M.; Ryden, A.; Ahlqvist, M.; Sunden, M.; Karlson, B. W.; Xu, Y.; Guo, J.; Mei, X.; Han, L.; Wang, J.; Li, J.; Shen, Z.	2017	Wrong outcomes - has some statistical data but not enough information regarding the intervention
Duenas, Maria; Sow, Sire; Wajnberg, Ania; Ma, Yixuan; Fuccillo, Mike	2019	Wrong outcomes: no full text found, conference abstract only
Estelrich, Maria Margalida Santandreu; Massanet, Antonio Palomero; Delgado, Olga; Sanchez-Raga, Jose Maria; Sureda, Bernat Galmes; Canaro, Mariana; Sampol, Antonia	2018	Not enough statistical detail
Faujdar, Dharamjeet S.; Sahay, Sundeep; Singh, Tarundeep; Kaur, Manmeet; Kumar, Rajesh	2020	Not enough statistical detail
Fioravanti, A.; Fico, G.; Arredondo, M. T.; Leuteritz, J. P.	2011	Wrong study design
Fioravanti, Alessio; Fico, Giuseppe; Salvi, Dario; GarcÃ­a-Betances, Rebeca; Arredondo, Maria; GarcÃ­a-Betances, Rebeca I.; Arredondo, Maria Teresa	2015	Wrong outcomes
Fischer, N.; Agboola, S.; Palacholla, R.; Atif, M.; Jethwani, K.; Kvedar, J.	2018	Protocol only
Fleming, James N.; Gebregziabher, Mulugeta; Posadas, Aurora; Su, Zemin; McGillicuddy, John W.; Taber, David J.	2021	Not enough statistical detail
Fortmann, Addie L.; Philis-Tsimikas, Athena; Bagsic, Samantha R. S.; Vital, Daniela G.; Jones, Jennifer A.; Sandoval, Haley; Savin, Kimberly L.; Clark, Taylor; Luu, Kimberly; Godino, Job G.; Gallo, Linda	2020	Wrong outcomes
Fortmann, Addie L.; Philis-Tsimikas, Athena; Bagsic, Samantha R. S.; Vital, Daniela G.; Jones, Jennifer A.; Sandoval, Haley; Savin, Kimberly L.; Clark, Taylor; Luu, Kimberly; Godino, Job G.; Gallo, Linda	2020	Duplicate
Frias, Juan; Virdi, Naunihal S.; Raja, Praveen; Kim, Yoona; Savage, George; Unger, Jeffrey; Raikhel, Marina; Osterberg, Lars	2016	Not enough statistical detail
Gomis-Pastor, Mar; Mirabet Perez, Sonia; Roig Minguell, Eulalia; Brossa Loidi, VicenÃ§; Lopez Lopez, Laura; Ros Abarca, Sandra; Galvez Tugas, Elisabeth; Mas-Malagarriga, NÃ°ria; Mangues Bafalluy, MÂª Antonia	2021	Not enough detail regarding the intervention
Gomis-Pastor M, Mirabet S. Roig E. Brossa V. Lopez L. Ros S. Mas N. Mangues M. A.	2020	Not enough statistical detail
Gondalia, R.; Kaye, L.; Barrett, M. A.; Stempel, D. A.	2021	Wrong outcomes
Gondalia, Rahul; Kaye, Leanne; Stempel, David; Barrett, Meredith; Van Sickle, David	2020	Not enough statistical detail
Gondalia, R.; Theye, B.; Stempel, D. A.	2019	English version of text not available
Harada, Norihiro; Harada, Sonoko; Ito, Jun; Atsuta, Ryo; Hori, Satoshi; Takahashi, Kazuhisa	2020	Wrong outcome (barriers to med adherence, not actual adherence)
Haridy, James; Iyngkaran, Guru; Nicoll, Amanda; Muller, Kate; Ramachandran, Jeyamani; Altus, Rosalie; Stewart, Jeff; Valaydon, Zina; Davies, Jane; Wilson, Mark; Colman, Anton; Nelson, Renjy; Tse, Edmund; Watkinson, Sally; Pronk, Daniel; Edgell, Cameron; Liew, Danny	2020	Not enough statistical detail
Heiney, Sue P.; Donevant, Sara B.; Adams, Swann Arp; Parker, Pearman D.; Chen, Hongtu; Levkoff, Sue	2020	Wrong outcomes
Heisler, Michele; Choi, Hwajung; Mase, Rebecca; Richardson, Caroline; Fagerlin, Angela; Palmisano, Gloria; Montori, Victor M.; Spencer, Michael; An, Laurence C.	2014	Wrong intervention - delivered by community health workers
Heisler, Michele; Choi, Hwajung; Palmisano, Gloria; Mase, Rebecca; Richardson, Caroline; Fagerlin, Angela; Montori, Victor M.; Spencer, Michael; An, Laurence C.	2014	Wrong intervention - involves community care worker involvement
Hendershot, C. S.; Dermody, S. S.; Wardell, J. D.; Stoner, S. A.	2017	Wrong comparator - control condition not usual care
Hixson, John	2016	Wrong intervention/outcomes
Hofmann, V.; Riese, C.; Schinkothe, T.	2018	Describes an intervention but is not an original research article
Howren, Alyssa; Tsao, Nicole; De Vera, Mary; Shojania, Kamran; Kydd, Alison; Friesen, Russell	2017	Wrong intervention
Howren, A.; Tsao, N.; De Vera, M. A.; Choi, H.; Kydd, A.; Shojania, K.; Friesen, R.	2017	Duplicate
Howren, Alyssa; Tsao, Nicole; Shojania, Kamran; Avina-Zubieta, Antonio; Kydd, Alison; De Vera, Mary; Choi, Hyon; Friesen, Russell	2018	Not enough statistical detail
Jprn, Umin	2021	Wrong patient population - includes adolescents
Kamal, Ayeesha Kamran; Khalid, Wardah; Zulfiqar, Maryam; Muqeet, Abdul; Zaidi, Fabiha; Gowani, Ambreen; Virani, Salim S.	2019	Not enough statistical detail
Katz, Richard J.; Nunlee-Bland, Gail; Magee, Michelle F.; Young, Heather; Witkin, Linda; Nassar, Carine; Cohen, Joshua L.	2019	Not enough statistical detail
Kaye, L.; Gondalia, R.; Remmelink, E.; Barrett, M.; Su, J.; Thompson, A.	2017	Not enough statistical detail
Kim, Chul-Gyu; Park, Hyeoun-Ae	2020	Wrong outcomes
Kleinman, Nora J.; Shah, Avani; Shah, Sanjiv; Phatak, Sanjeev; Viswanathan, Vijay	2011	English version of text not available
Krackhardt, Markus Florian; Maier, Lars; Jornten-Karlsson, Magnus	2017	English version of text not available
Krackhardt Mf, Maier L. Jornten-Karlsson M.	2019	Not enough statistical detail
Kravitz, Richard L.; Marois, Maria; Schmid, Christopher H.; Wang, Youdan; Wilsey, Barth; Ward, Deborah; Hays, Ron D.; Duan, Naihua; Macdonald, Scott; Jerant, Anthony; Servadio, Joseph L.; Haddad, David; Sim, Ida	2019	Protocol only
Krishnan, Archana; Weikum, Damian; Cravero, Claire; Kamarulzaman, Adeeba; Altice, Frederick L.	2018	Wrong intervention - used patient/physician dyads and had regular reminder calls from physicians
La Grange, Ricardo; Lewis, Maria; Rockwern, Brooke	2021	Wrong outcomes
Li, Shuang; Psihogios, Alexandra M.; McKelvey, Elise R.; Ahmed, Annisa; Rabbi, Mashfiqui; Murphy, Susan	2012	Wrong patient population
Linder, L.; Jo, Y.; Wu, Y.; Wilson, A.; MacPherson, C. F.; Johnson, R.	2020	Wrong patient population- adolescent and child related
Mburu, Stephen; Oboko, Robert	2018	Wrong population - paediatric population
McGillicuddv, J. W.; Sox, L.; Chandler, J.; Treiber, F.; Taber, D.	2018	Wrong outcomes
McGillicuddy, John; Anderson, Ashley; Favela, April; Sox, Luke; Wilder, Spencer; Brunner-Jackson, Brenda; Mueller, Martina; Treiber, Frank	2019	Not enough statistical detail
McGillicuddy, John; Sox, Luke; Brunner-Jackson, Brenda; Taber, David; Mueller, Martina; Chavin, Kenneth; Baliga, Prabakur; Treiber, Frank	2015	No control condition
Moczygemba, Leticia R.; Thurman, Whitney; Tormey, Kyler; Hudzik, Anthony; Welton-Arndt, Lauren; Kim, Elizabeth	2015	Not enough statistical detail
Mohr, David C.; Stiles-Shields, Colleen; Brenner, Christopher; Palac, Hannah; Montague, Enid; Kaiser, Susan M.; Carty-Fickes, Eric; Duffecy, Jenna	2021	Wrong outcomes
Moorhead, Penjit; Zavala, Ana; Kim, Yoona; Virdi, Naunihal S.	2015	Not enough statistical detail
Morak, Juergen; Schwarz, Mark; Hayn, Dieter; Schreier, Guenter	2017	Wrong intervention - usual care group not included in analysis
Muessig, Kathryn E.; LeGrand, Sara; Horvath, Keith J.; Bauermeister, JosÃ^©^ A.; Hightow-Weidman, Lisa B.	2012	Wrong outcomes
Muldoon, Matthew F.; Einhorn, Julian; Yabes, Jonathan; Burton, Danielle; Rollman, Bruce; Irizarry, Taya; Basse, Jeanne; Burke, Lora E.; Kamarck, Thomas; Suffoletto, Brian	2017	Wrong study design
Mulley, J.; Coorey, G.; Peiris, D.; Usherwood, T.; Redfern, J.; Neubeck, L.	2020	Not enough statistical detail
Nabaggala, Maria; Kiragga, Agnes; Naggirinya, Naggirinya Agnes; Oseku, Elizabeth; Akirana, Josephine; Oyat, Phillip; Pattery, Theresa; Ratanshi, Rosalind	2019	Not enough detail
Narasimhan, Padmanesan; Bakshi, Aishwarya; MacIntyre, Chandini Raina; Ray, Pradeep; Kittusami, Sathyapriya; Prashant, Suma; Mathai, Dilip; Bakshi, Kasturi	2019	Wrong outcomes
Naslund, John A.; Marsch, Lisa A.; McHugo, Gregory J.; Bartels, Stephen J.	2014	Wrong intervention
Nct,	2015	Duplicate
Nct,	2015	Wrong population (aged 14 years +)
Ngowi, Kennedy Michael; Lyamuya, Furaha; Mmbaga, Blandina T.; Muro, Eva; Hillu, Zawadiel; Shirima, Mary; Aarnoutse, Rob E.; Ag Sprangers, Mirjam; Nieuwkerk, Pythia T.; Reiss, Peter; Sumari-de Boer, Marion	2021	Not enough detail
O'Dwyer, Susan; Greene, Garrett; MacHale, Elaine; Cushen, Breda; Sulaiman, Imran; Boland, Fiona; Bosnic-Anticevich, Sinthia; Mokoka, Matshediso C.; Reilly, Richard B.; Taylor, Terence; Ryder, Sheila A.; Costello, Richard W.	2020	Wrong outcomes
Ogarkov, O.; Hodges, J.; Zhdanova, S.; Koshkina, O.; Suzdalnitsky, A.; Moiseeva, E.; Waldman, A. L.; Koshcheev, M.; Schwendinger, J.; Vitko, S.; Dillingham, R.; Heysell, S.	2020	Wrong intervention
Okuboyejo, Senanu; Eyesan, Omatseyin	2020	Wrong outcomes
Osterberg, Lars; Virdi, Naunihal; Kim, Yoona; Raja, Praveen; Cruz, Miriam; Savage, George; Unger, Jeffrey; Raikhel, Marina; Frias, Juan	2014	Describes an intervention but is not an actual study research article
Ovbiagele, Bruce; Jenkins, Carolyn; Patel, Sachin; Brunner-Jackson, Brenda; Anderson, Ashley; Saulson, Raelle; Treiber, Frank	2016	Wrong outcomes
Pandey, Avinash; Choudhry, Niteesh	2015	Wrong outcomes
Parkes-Ratanshi, Rosalind M.; Nabaggala, Maria S.; Bwanika, Agnes N.; Lamorde, Mohammed; King, Rachel; Owarwo, Noela; Odongpiny, Eva A. Laker; Orama, Richard; Castelnuovo, Barbara; Kiragga, Agnes	2015	Wrong outcomes
Patterson, Jonathan; Allison, Matt; Brassil, Kelly J.; Burton, Stephen B.; Norbu, Thupten T.; Rasulnia, Mazi; Patel, Dhiren	2019	Wrong outcomes
Pedersen, Natalia	2020	Wrong study design
Pidermann, B.; Park, H.; Bijlani, A.; Travis, E.; Truscott, A.; Purvance, C.	2015	Wrong study design
Piette, John D.; Striplin, Dana; Marinec, Nicolle; Chen, Jenny; Trivedi, Ranak B.; Aron, David C.; Fisher, Lawrence; Aikens, James E.	2017	Wrong study design - no control condition
Prabhakaran, Dorairaj; Singh, Kavita; Kondal, Dimple; Jacob, Pramod; Goenka, Shifalika; Jha, Dilip; Gupta, Priti; Ajay, Vamadevan S.; Jindal, Devraj; Prieto-Merino, David; Roy, Ambuj; Perel, Pablo; Tandon, Nikhil; Patel, Vikram	2015	Wrong patient population and design - intervention required input from carers
Pugh, Steven; Patel, Yashma; Morrow, Thomas	2018	Not enough statistical detail
Ramsey, Susan E.; Ames, Evan G.; Uber, Julia; Habib, Samia; Clark, Seth; Waldrop, Drenna	2017	Not enough detail
Rohde, Jacob A.	2021	Wrong intervention - intervention includes a face to face session with a physio
Rosen, Craig S.; Azevedo, Kathryn J.; Tiet, Quyen Q.; Greene, Carolyn J.; Wood, Amanda E.; Calhoun, Patrick; Bowe, Thomas; Capehart, Bruce P.; Crawford, Eric F.; Greenbaum, Mark A.; Harris, Alex H. S.; Hertzberg, Michael; Lindley, Steven E.; Smith, Brandy N.; Schnurr, Paula P.	2021	No statistical detail
Sarfo, Fred Stephen; Treiber, Frank; Gebregziabher, Mulugeta; Adamu, Sheila; Nichols, Michelle; Singh, Arti; Obese, Vida; Sarfo-Kantanka, Osei; Sakyi, Asumadu; Adu-Darko, Nyantakyi; Tagge, Raelle; Agyei-Frimpong, Marian; Kwarteng, Naomi; Badu, Elizabeth; Mensah, Nathaniel; Ampofo, Michael; Jenkins, Carolyn; Ovbiagele, Bruce	2017	Wrong intervention
Sarfo Fs, Treiber F. Gebregziabher M. Adamu S. Nichols M. Singh A. Obese V. Sarfo-Kantanka O. Sakyi A. Adu-Darko N. Tagge R. Agyei-Frimpong M. Kwarteng N. Badu E. Mensah N. Ampofo M. Jenkins C. Ovbiagele B.	2019	Wrong intervention - under nurse guidance
Sarzynski, Erin; Decker, Brian; Thul, Aaron; Weismantel, David; Melaragni, Ronald; Cholakis, Elizabeth; Tewari, Megha; Beckholt, Kristy; Zaroukian, Michael; Kennedy, Angie C.; Given, Charles	2018	Wrong intervention
Scholten, Mark R.; Kelders, Saskia M.; Van Gemert-Pijnen, Julia E. W. C.	2017	Wrong outcomes - does not statistically analyse diffs in adherence pre test to post test
Serlachius, Anna; Schache, Kiralee; Kieser, Anel; Arroll, Bruce; Petrie, Keith; Dalbeth, Nicola	2021	Wrong outcomes
Sheng, Tong; Kanchi, Arati; Parks, Linda; Bailey, Timothy; Greenfield, Michael S.	2019	Wrong outcomes and not enough data
Sloots, Joanne; Bakker, Mirthe; Eijsvogel, Michiel; van der Valk, Paul; van Ommeren, Clara; van der Palen, Job; Linssen, Gerard; Grinovero, Martijn; Tabak, Monique; Effing, Tanja; Lenferink, Anke	2018	Wrong outcomes
Sunil Kumar, D.; Prakash, B.; Thomas, Jose Jom; Kulkarni, Praveen; Gopi, Arun; Murthy, M. R. Narayana; Subhash Chandra, B. J.; Kadkol, Padma Shrinivas; Arun, Vanishri	2021	No control condition and wrong outcomes
Sutton, Reed T.; Wierstra, Kelsey; Huang, Vivian W.	2021	Wrong outcomes
Takizawa, Makiko; Kawamoto, Fumiko; Terunuma, Noriko; Ueki, Jun; Tobino, Kazunori; Fukuchi, Yoshinosuke	2018	Wrong comparator
Tricarico, Christopher; Peters, Robert; Som, Avik; Javaherian, Kavon; Ross, Will	2009	Wrong outcomes
Twimukye, Adelline; Bwanika Naggirinya, Agnes; Kasirye, Ronnie; Kiragga, Agnes; Castelnuovo, Barbara; Wasswa, Jacob; Nabaggala, Maria Sarah; Lamorde, Mohammed; Parkes-Ratanshi, Rosalind; Katabira, Elly; King, Rachel Lisa	2017	Wrong outcomes
Virdi, Naunihal S.; Kim, Yoona; Raja, Praveen; Savage, George; Osterberg, Lars	2021	Wrong study design
Voncken-Brewster, Viola; Tange, Huibert; Moser, Albine; Nagykaldi, Zsolt; de Vries, Hein; van der Weijden, Trudy	2016	Not enough statistical detail
Wang, Hongyi; Zhang, Jing; Chu, Zhenxing; Hu, Qinghai; Dong, Willa; Huang, Xiaojie; Chen, Yaokai; Wang, Hui; He, Xiaoqing; Zhang, Lukun; Hu, Zhili; Bao, Rantong; Li, Shangcao; Li, Hang; Cui, Sitong; Jin, Xia; Ding, Haibo; Geng, Wenqing; Jiang, Yongjun; Xu, Junjie	2014	Wrong outcomes
Ward, Richard; Taha, Karim M.	2020	Wrong intervention
Wu, Yelena P.; Kanokvimankul, Patsaporn; Fowler, Brynn; Parsons, Bridget G.; Linder, Lauri A.; Macpherson, Catherine F.; Johnson, Rebecca H.	2016	Wrong study design - no control condition and no statistical outcome data
Yasmin, F.	2018	Wrong patient population - includes adolescents
Zha, Peijia; Qureshi, Rubab; Porter, Sallie; Chao, Ying-Yu; Pacquiao, Dula; Chase, Sabrina; O'Brien-Richardson, Patricia	2015	Wrong intervention - interactive voice calls
	2020	Wrong outcomes - reports on med adherence self-efficacy

### Appendix II: Critical appraisal

**Table 2.** Effective Public Health Practise Project (EPHPP) Quality Assessment Tool for the appraisal of included studies.

Study	Selection bias	Study design	Confounders	Blinding	Data collection methods	Withdrawals and drop-outs	Global rating
Weitzman (2021)	Weak	Moderate	Moderate	Moderate	Weak	Weak	Weak
Vluggen (2021)	Moderate	Strong	Strong	Moderate	Strong	Weak	Moderate
Schoenthaler (2020)	Strong	Strong	Moderate	Moderate	Strong	Strong	Strong
Sarfo (2018)	Moderate	Strong	Strong	Weak	Weak	Strong	Weak
Ni (2018)	Moderate	Strong	Strong	Moderate	Strong	Weak	Moderate
McGillicuddy (2013)	Moderate	Strong	Weak	Moderate	Strong	Strong	Moderate
McClure (2016)	Strong	Strong	Weak	Moderate	Strong	Strong	Moderate
Magnani (2017)	Moderate	Moderate	Strong	Moderate	Strong	Weak	Moderate
Lee (2015)	Moderate	Moderate	Strong	Weak	Strong	Strong	Moderate
Kidd (2019)	Weak	Moderate	Weak	Weak	Strong	Strong	Weak
KamalAk (2016)	Weak	Strong	Strong	Moderate	Strong	Strong	Moderate
Dale (2015)	Moderate	Strong	Weak	Weak	Moderate	Strong	Moderate
Buis (2017)	Moderate	Strong	Moderate	weak	Strong	Strong	Moderate
Arora (2014)	Moderate	Strong	Weak	Moderate	Strong	Moderate	Moderate

### Appendix III: Characteristics of included studies

**Table 3.** Study characteristics.

Study	Country	Study aim	N	Age M (*SD*)	Gender	Illness status	Duration	Platform	Intervention aim	Outcome measure	Study design
Magnani (2017)	United States	To pilot a smartphone-based relational agent as preparation for a randomised clinical trial	31	68 (11)	61% male	Atrial fibrillation	30 days	Mobile app	To serve as a health counsellor or coach-specific to AF	Self-report – Morisky Medication Adherence Scale (MMAS)-8	Cohort study
Kidd (2019)	Canada	To examine A4i use over a 1-month period using a pre–post, multiple-method design	38	31.42 (range 19–61)	71% male	Primary schizophrenia diagnosis (*n* = 24), schizoaffective (*n* = 9) and the remaining having psychosis NOS or psychosis comorbid with bipolar disorder (*n* = 1) and autism with prominent psychosis symptomatology (*n* = 1).	25 days	Mobile app	To use feed scheduling, and text-based functions co-designed with service users to enhance illness self-management	Self-report – Brief Adherence Rating Scale	Cohort study
Lee (2015)	United States	To test the feasibility of mobile applications for seniors to enhance Safe anticoagulation therapy (MASS)	18	67.28 (8.72)	78% male	Atrial fibrillation, heart failure or those at risk for venous thromboembolism	3 months	Mobile app	To promote independence and self-care	Self-report – Morisky Medication Adherence Scale (MMAS)	Non-randomised experimental study
Weitzman (2021)	United States	To test the acceptability, usability and clinical impact of the Dot app.	54	25.6 (2.27)	100% male	HIV	6 weeks	Mobile app	Self-management and medication adherence	Self-report – adapted 3-item HIV anti-retroviral treatment adherence measure	Cohort study
Vluggen (2021)	Netherlands	To examine the effectiveness of an eHealth program on overall treatment adherence	478	60 (*SD*?)	68% male	Diabetes Type 1 and Type 2	6 months	Web-based	Self-management and medication adherence	Self-report – measured with the Probabilistic Medication Adherence Scale (ProMAS) questionnaire. For OHA adherence and an adapted version of the ProMAS. For insulin therapy adherence	RCT
Schoenthaler (2020)	United States	To develop and evaluate the acceptability and preliminary efficacy of an mHealth adherence intervention on medication adherence, systolic BP, diastolic BP and HbA1c in Black patients with uncontrolled HTN and/or T2D	42	57.6 (11.1)	55% male	Uncontrolled HTN and/or T2D	3 months	Web portal	Self-management and medication adherence	Self-report – 8 item MMAS	RCT
Sarfo (2018)	Ghana	To test the feasibility and preliminary efficacy of an m-Health technology-enabled intervention in improving BP control among Ghanaian stroke patients within 1 month of symptom onset	60	55 (13)	65% male	Strokes	3 months	Text messaging platform	Self-management	Self-report and medication possession ratio recorded via the app	RCT
Ni (2018)	China	To develop a mobile technology intervention to improve medication adherence among patients with coronary heart disease (CHD)	50		80.6% male	CHD	30 days	Text messaging platform	Medication adherence	Self-report – Voils Medication Non-Adherence Extent Scale	RCT
McGillicuddy (2013)	United States	To assess the feasibility, acceptability and preliminary outcomes of a prototype mobile health medication and BP self-management system for kidney transplant patients with uncontrolled hypertension	19	Intervention *M* (*SE*) 42.44 (12.04), control 57.6 (8.28)	38% male	Kidney transplant recipients with uncontrolled HPT	3 months	Electronic meds tray plus text messaging	Self-management and medication adherence	Objective – electronic medication tray	RCT
McClure (2016)	United States	To assess feasibility and acceptability of the My Mobile Advice smoking cessation program (MyMAP) and estimate its effects on smoking cessation and medication adherence to inform future research planning	66	49.5 (8.7)	44% male	smokers	5 months	Web and Mobile app	Smoking cessation and medication adherence	Objective and self-report – Varenicline use was assessed using automated pharmacy fill data and self-reported days of use. Objective data was not reported separately but is combined with self-report to estimate adherence.	RCT
KamalAk (2016)	Pakistan	To develop and test the effectiveness of a tailored health information technology driven intervention: Talking Prescriptions (Talking Rx) to improve medication adherence in a resource challenged environment	197	Intervention 59.1 (11.6), control 57.7 (11.1)	77% male	History of CVA or CAD	3 months	Mobile app	To boost medication adherence and medication health literacy	Self-report – 8-item Morisky Medication Adherence scale 8 (MMAS-8)	RCT
Dale (2015)	New Zealand	To investigate the effectiveness of a mHealth-delivered comprehensive CR program (Text4Heart) to improve adherence to recommended lifestyle behaviours (smoking cessation, physical activity, healthy diet and nonharmful alcohol use)	123	59.5 (*SD* 11.1)	81% male	CHD	6 months	Text messaging platform	The aim was to mirror current CR programs in educating patients about their cardiovascular risk factors and supporting them to make relevant lifestyle changes	Self-report – Morisky 8-item Medication Adherence Questionnaire	RCT
Buis (2017)	United States	To determine the feasibility, acceptability and preliminary clinical effectiveness of BPMED, an intervention designed to improve medication adherence among African Americans with uncontrolled HTN	123	49 (*SD* 8.3)	44% male	Hypertension	1 month	Text messaging platform	To improve medication adherence through reminders and educational material	Self-report – Morisky Medication Adherence Scale (MMAS)	RCT
Arora (2014)	United States	To evaluate a broadly scalable mHealth intervention (health-related daily text messages for 180 days) for resource-poor ED patients with diabetes	128	50.7 (10.2)	31% male	Diabetics with HbA1c level greater than/equal to 8%	6 months	Text messaging platform	To enhance patient motivation, self-efficacy and ability to perform diabetes self-care behaviours and medication adherence	Self-report – Morisky 8-item Medication Adherence Questionnaire	RCT

AF: Atrial fibrillation; BP: blood pressure; CAD: coronary artery disease; CHD: coronary heart disease; CR: cardiac rehabilitation; CVA: cerebrovascular accident; ED: emergency department; HbA1c: haemoglobin A1c; HIV: human immunodeficiency virus; HTN: hypertension; MMAS-8: Morisky 8-item Medication Adherence Scale; NOS: not otherwise specified; OHA: oral hypoglycemic agent; RCT: randomised controlled trial; SD: standard deviation; SE: standard error; T2D: type 2 diabetes.
